# Bio-inspired silver selenide nano-chalcogens using aqueous extract of *Melilotus officinalis* with biological activities

**DOI:** 10.1186/s40643-021-00412-3

**Published:** 2021-07-02

**Authors:** Seyedeh Zahra Mirzaei, Hamed Esmaeil Lashgarian, Maryam Karkhane, Kiana Shahzamani, Alaa Kamil Alhameedawi, Abdolrazagh Marzban

**Affiliations:** 1 Razi Herbal Medicines Research Center, Lorestan University of Medical Sciences, P.O. Box: 6816889468, Khorramabad, Iran; 2 Biotechnology Department, Faculty of Medicine, Lorestan University of Medical Sciences, Khorramabad, Iran; 3 Isfahan Gastroenterology and Hepatology Research Center (IGHRC), Isfahan University of Medical Sciences, Isfahan, Iran; 4 Iraqi Ministry of Education, Baghdad, Iraq

**Keywords:** Silver selenide nano-chalcogens (Ag_2_Se-NCs), *Melilotus officinalis*, *Pseudomonas aeruginosa*, Biological activities

## Abstract

For the first time, an aqueous extract of *Melilotus officinalis* was used to synthesize bimetallic silver selenide chalcogenide nanostructures (Ag_2_Se-NCs). The formation of NCs was confirmed and characterized by UV–visible and FTIR spectroscopy, SEM and TEM imaging, XRD and EDX crystallography, zeta potential (ZP) and size distribution (DLS). The bioactivities of biosynthesized Ag_2_Se-NCs, such as antibacterial, antibiofilm, antioxidant and cytotoxicity potentials, were then examined. Bio-based Ag_2_Se-NCs were successfully synthesized with mostly spherical shape in the size range of 20–40 nm. Additionally, the MIC and MBC values of Ag_2_Se-NCs against β-lactam-resistant *Pseudomonas aeruginosa* (ATCC 27853) were 3.12 and 50 µg/ml, respectively. The DPPH scavenging potential of Ag_2_Se-NCs in terms of IC_50_ was estimated to be 58.52. Green-synthesized Ag_2_Se-NCs have been shown to have promising benefits and could be used for biomedical applications. Although the findings indicate promising bioactivity of Ag_2_Se-NCs synthesized by *M. officinalis* extract (*MO*), more studies are required to clarify the comprehensive mechanistic biological activities.

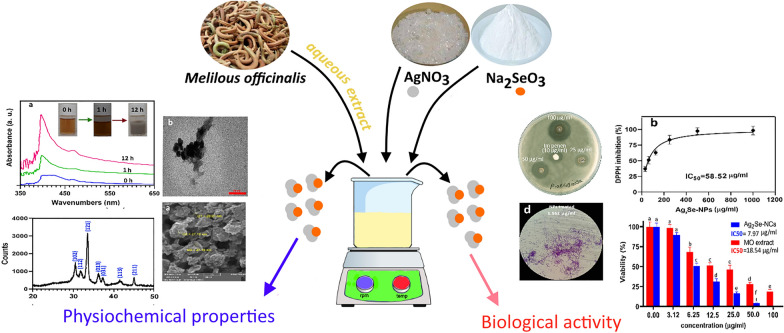

## Introduction

Silver chalcogenides, particularly Ag_2_Se, are semiconductors with impressive physicochemical properties being used in electronics, optical conductors, infrared detectors, electromagnetic field sensors and optical filters. A variety of studies have examined the different properties of Ag_2_Se on the scale of microstructures, nanostructures, quantum dots and bulk forms (Martinez-Nuñez et al. 2016; Vo et al. 2016). Ag_2_Se nanostructures, in particular quantum dots, have unique properties that make them suitable for bioimaging (Yang et al. 2018). Generally, Ag_2_Se-NCs can be synthesized in two distinct crystalline phases. Orthorhombic crystalline phase (β-Ag_2_Se) have photocatalytic and fluorescence-emitting activities used in the manufacture of optical sensors and light-sensitive films. In contrast, the body-centered cubic phase (α-Ag_2_Se) is a metallic structure with electrolyte properties mainly used to manufacture batteries (Ayele 2016).

The most common processes, including high-temperature synthesis, microwave irradiation, electrochemical method and sonochemical reaction, have been developed for the synthesis of Ag_2_Se-NCs (Jafari et al. 2013). These techniques have limitations due to the use of chemical reagents, various solvents and increased energy consumption. Many researchers have also attempted alternative and more accessible approaches to achieve Ag_2_Se nano-chalcogenides (Ayele 2016; Gholami et al. 2018; Sibiya and Moloto 2017; Yang et al 2018).

Several different approaches have been established for the use of bioactive compounds for nanoparticles (NPs) synthesis (Gilavand et al. 2021). Biological processes using natural reactants such as plant metabolites and microorganisms have been found cheaper and more reliable. However, NPs are inherently unstable due to an increased van der Waals force on their surface that tends to aggregate (Liu et al. 2018). In this regard, biologically active metabolites such as polyphenols, flavonoids, terpenoids and biopolymers such as nucleic acids, lipids and proteins are excellent capping agents that prevent them from accumulating as well as preventing their uncontrolled growth (Dobrucka 2020).

One of the most important applications of biosynthesized NPs is the development and design of pharmaceutical formulations. Since the antimicrobial potential of NPs is well established, it can be an attractive strategy for controlling bacterial, fungal, viral and parasitic infections (Lu et al. 2020). On the other hand, new antimicrobial drugs are required due to the advent of microbial resistance. *M. officinalis* is a medicinal plant with extensive therapeutic properties. Several metabolites of *M. officinalis* with remarkable biological activities are found that could be used for bioreduction of metal in the nanoparticle synthesis. According to the studies, some major bioactive compounds are coumarins derivatives, hydroxycinnamic glucosides, phenolic acids, *p*-hydroxybenzoic acid, chlorogenic acid, vanillic acid, caffeic acid, salicylic acid, ferulic acid and ellagic acid (Anthony 2009; Kanipandian and Thirumurugan 2014; Mirzaei et al. 2020)*.* Although various properties of Ag_2_Se-NCs have been reported, their biological properties have not been studied by researchers (Gopinath et al. 2013; Liu et al 2018; Sibiya and Moloto 2017). In this study, an aqueous extract of *M. officinalis* (*MO* extract) was used as a reducing and capping agent for the synthesis of Ag_2_Se-NCs. After that, the antibiofilm, antibacterial, antioxidant and cytotoxicity properties of biosynthesized NCs were investigated.

## Materials and methods

### Materials and reagents

All materials were provided from Sigma chemical company (St. Louis, MO). Microbial culture media were purchased from Himedia (Mumbai, India). Human hepatocellular carcinoma, HepG2 (ATCC HB-8065) cell line was prepared from the cell collection bank of the Pasteur Institute of Iran. The bacterial strain, *Pseudomonas aeruginosa* (ATCC 27853) was procured from the IROST microbial culture collection (Tehran, Iran).

### Aqueous extraction and phytochemical analyses

#### Aqueous extract preparation and flavonoid detection

Dried *M. officinalis* seeds were purchased from Attarak online market for herbal medicines, Tehran, Iran. After washing the seeds, 10 g was added to 100 ml of distilled water and placed in a bath-sonicator at 60 °C for 30 min. The extract was then filtered using a Whatman filter (No. 1). The presence of phytochemicals was qualitatively detected as described in the previous study. To detect the flavonoid contents, aluminum nitrate solution (10% W/V in methanol) and lead acetate (0.1% W/V) were gently dropped into the *MO* extract. The appearance of yellow color indicates flavonoids’ presence in the sample solution.

#### Total terpenoids detection

Total terpenoids were assayed by mixing 2 ml of chloroform with 5 ml of *MO* extract and 3 ml of sulfuric acid 98%. The formation of a reddish-brown ring interface of the solution indicated the presence of terpenoids.

#### Total glycosides detection

Two types of glycosides were examined in the *MO* extract. First, a ratio of acetic acid: chloroform (1:1 V/V) was mixed with *MO* extract and vortexed. After that, 1 ml of sulfuric acid 98% was added and the change of mixture color to green was monitored. The second, a ratio of 10 to 0.5 ml of acetic acid: FeCl_3_ (2% W/V) was mixed with 5 ml of *MO* extract. After a moment, with adding 1 ml of sulfuric acid 98%, the appearance of a brown ring between the phase layers indicated cardiac steroidal glycosides in the *MO* extract.

#### Total phenols and saponin detection

Total phenolic compounds were detected by adding ferric chloride solution 1% to the *MO* extract and the appearance of bluish-black color indicated the presence of phenols. To detect saponins, 5 mg of *MO* extract was added to 10 ml of distilled water in a 15-ml Falcon tube and vigorously shaken until the formation of a foam layer over the solution.

### Biosynthesis of Ag_2_Se-NCs

To fabricate Ag_2_Se-NCs, the filtrated extract was used as reducing and capping agent. For this purpose, 10 ml of the filtrate was poured into 100-ml flasks containing 40 ml of deionized water (DW). Then 1 ml of silver nitrate (AgNO_3_) (1 mM) and 0.5 ml of sodium selenite (Na_2_SeO_3_) (0.45 mM) solutions were added to the mixture under shaking on a magnetic stirrer for 12 h. The mixture was sonicated with an ultrasonic horn by 50 kHz for 5 min. The gray suspension was centrifuged at 13,000 rpm at 4 °C, washed twice by ethanol 96% and DW. Finally, the precipitate was dried at 60 °C in an oven for 24 h.

### Characterization of Ag_2_Se-NCs

After the synthesis of Ag_2_Se-NCs, UV–visible spectroscopy was performed using a spectrophotometer (Jenway UV–Vis, 6505 model, UK) at the range of 200–800 nm. FTIR spectrometer (Bruker IFS 66/s, Bruker Optics, Billerica, MA) was applied to study functional groups involved in NPs formation. Transmission electron microscopy (TEM) micrographs were taken with a TEM instrument (Philips EM 208S, Netherlands). Morphological and elemental properties were studied through SEM and energy dispersive X-ray (EDX) analyses using FE-SEM TESCAN MIRA3. X-ray diffraction (XRD) of biosynthesized Ag_2_Se-NCs was carried out using an XRD diffractometer (Rigaku Ultima IV) in the normal angle range (10–80 °C). The dynamic Light Scattering (DLS) and zeta potential (ZP) of Ag_2_Se-NCs were determined by a DLS instrument (VASCO, CORDOUAN TECHNOLOGIES, England).

### Preparation of Ag_2_Se-NCs for bioassay experiments

The dry powder of Ag_2_Se-NCs was weighed and then dispersed in distilled water in the desired amounts. The Ag_2_Se-NCs mixture was placed in a bath-sonicator for 1 h to achieve high homogeneity and reduce aggregation. After that, the suspension was then passed through a 0.45-μm filter paper and the filtrate was applied for biological assays.

### Antimicrobial assay of Ag_2_Se-NCs

The antimicrobial activity of biosynthesized Ag_2_Se-NCs was carried out by the agar well-diffusion method against βL-resistant *P. aeruginosa*. Imipenem (10 µg/ml) was used as a positive control for bacteria. Bacterial cells were spread on the agar plates using sterile swab; then, 20 µl of different concentrations of Ag_2_Se-NCs (1.5–100 µg/ml) were added to each well. After 24 h incubation at 37 °C, the growth inhibition zone was measured using a ruler.

Minimum inhibitory concentration (MIC) was determined using the micro-dilution method in 96-well plates. Fifty microliter of Ag_2_Se-NCs dilutions (0–100 µg/ml) were added to 50 µl of Muller–Hinton broth (MHB) containing 10^6^ CFU/ml of bacterial cells. After 24 h, the MIC value of Ag_2_Se-NCs was determined in terms of triphenyl tetrazolium chloride (TTC) reduction rate. Minimum bactericidal concentration (MIC) was obtained from MIC value as described previously (Sibiya and Moloto 2016).

### Antibiofilm activity

Biofilm formation of *P. aeruginosa* was studied in the presence of Ag_2_Se-NCs sub-MIC in a 96-well plate. Briefly, 10^6^ CFU/ml of bacterial cells were added to 200 μL of LB broth medium supplemented with 1.56 and 3.12 µg/ml of NPs. After 24 h incubation, non-adherent bacterial cells were removed. The adhered biofilms were stained with 0.1% crystalline violet solution for 5 min. Excess colors was then removed using DW and the biofilm was destained with 200 µl glacial acetic acid 35%. After that, the plate was slowly agitated for 2 min and the absorbance was determined by a microplate reader at 570 nm. The following equation (Eq. 1) was used to determine the antibiofilm efficacy of Ag_2_Se-NCs:

1 $$ {\text{Antibiofilm}}\;{\text{efficacy}}\left( \%  \right) = \frac{{{\text{Control}}\left( {{\text{OD}}} \right) - {\text{treated}}\left( {{\text{OD}}} \right)}}{{{\text{control}}\left( {{\text{OD}}} \right)}} \times 100. $$

The adhesion ability of the bacterial biofilm was visualized by a qualitative method. Briefly, the bacterial cells were cultured in broth media supplemented with certain concentrations of Ag_2_Se-NCs, (25 and 50% of MIC) in contact with glass slides. After that, the bacterial biofilms were stained with 200 µl of crystal violet (1% w/v). The slides were then rinsed with 70% ethanol, washed by DW and dried at room temperature. Finally, the biofilms were photographed under a light microscope. Further, 3D topographical studies of treated and untreated biofilms were studied using atomic force microscopy (AFM).

### Antioxidant assays

The antioxidant capacities of Ag_2_Se-NCs and *MO* extract were examined using DPPH radical scavenging method. Briefly, different concentrations (10–1000 μg/ml) of Ag_2_Se-NCs and *MO* extract were added to methanol solution of DPPH reagent (0.1 mM) and remained for 30 min in darkness. The ascorbic acid was used as a positive control and ethanol as a blank in this assay. The scavenging percentage of DPPH was calculated by the following equation:

2 $$ {\text{Scavenging}}\;{\text{efficacy}}\left( \%  \right) = ~\frac{{{\text{Blank}}\left( {{\text{A}}0} \right) - {\text{Sample}}\left( {\text{A}} \right)}}{{{\text{Blank}}\left( {{\text{A}}0} \right)}}~~ \times 100. $$

### Cytotoxicity assay

Cellular toxicity of Ag_2_Se-NCs and *MO* extract against HepG2 cell line was studied using the MTT assay method. For this, the cells (1 × 10^4^ cells/well) were grown in a 96-well plate containing RPMI supplemented with 10% FBS, penicillin (100 IU/l) and streptomycin (100 mg/l) and incubated at 37 °C under 5% CO_2_ and 95% humidity conditions. After reaching about 75% confluence, the medium was refreshed with a medium containing serially diluted Ag_2_Se-NCs and the cells were again incubated for 24 h. After that, to each well, 100 µl of the MTT [3-(4, 5-dimethylthiozol-2-yl)-3,5-diphenyl tetrazolium bromide] (Sigma) solution was added and incubated at 37 °C for 4 h. Further, the medium containing unreduced MTT reagent was gently removed and the wells were treated with 200 µl of dimethyl sulfoxide (DMSO). To solubilize the formazan crystals, the plate was shaken for 15 min and then the absorbance solution was measured at 595 nm using an ELISA microplate reader. The viability of the cells was calculated by Eq. 2 as follows:

$$ {\text{Viability}}\left( \%  \right) = \frac{{{\text{Absorbance}}\;{\text{of}}\;{\text{treated}}\;{\text{sample}}\left( {\text{A}} \right)~}}{{{\text{Absorbance}}\;{\text{of}}\;{\text{control}}\left( {{\text{A}}0} \right)}} \times 100. $$

## Results and discussion

### Phytochemical analysis of MO extract

**Fig. 1:**
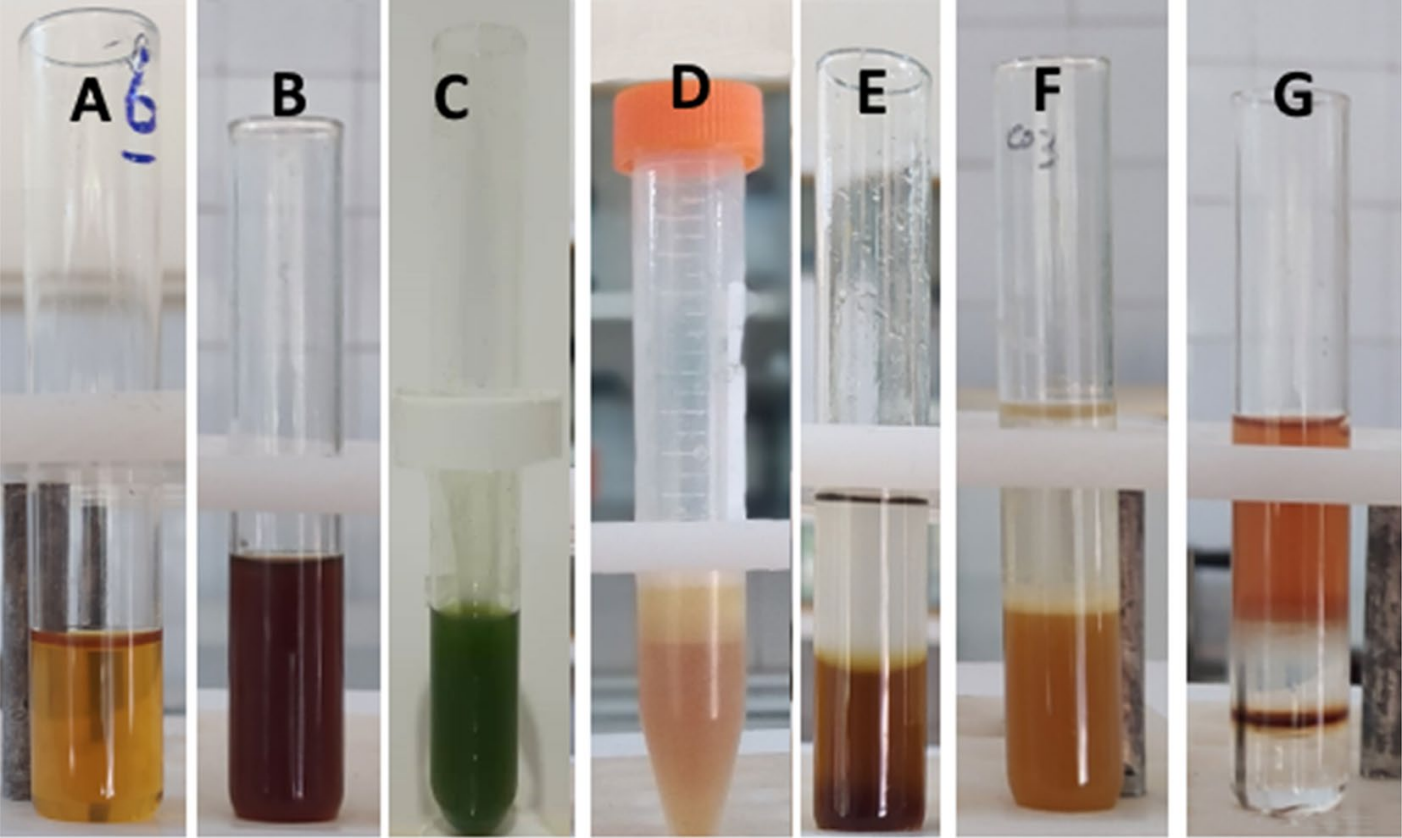
Qualitative assay of phytochemical contents in MO extract. **a** Flavonoids, **b** terpenoids, **c** aglycone steroidal glycoside, **d** saponin, **e** polyphenols, **g** cardiac steroidal glycosides

Phytochemical compounds in *MO* extract were quantitatively identified using standard methods based on colorimetric observations. As shown in Fig. 1, terpenoids, flavonoids, glycosides, polyphenols, and saponins were detected in *MO* extract. Many studies have previously demonstrated that *M. officinalis* contains major biologically active metabolites such as flavonoids, coumarins, steroid glycosides, saponins and other compounds (Liu et al 2018). Additionally, biological compounds responsible for biosynthesizing the most NPs include phenolic compounds, flavonoids, fatty acids, reducing sugars and polyhydric alcohols (Kanchi et al. 2020; Loeschner et al. 2011; Tripathy et al. 2020; Vorobyev et al. 2018). Therefore, the high potential of *MO* extract for the synthesis of various metal NPs can be attributed to these active metabolites.

### Ag_2_Se-NCs synthesis and characterization

**Fig. 2:**
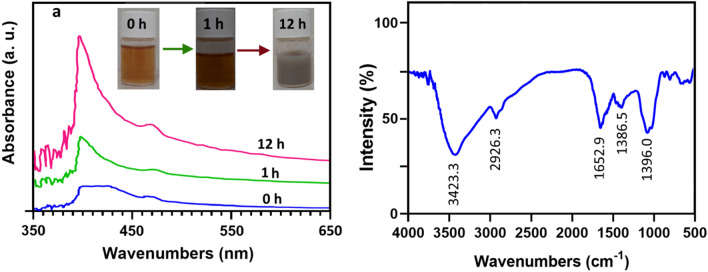
**a** UV–visible absorption and **b** FTIR spectra of Ag_2_Se-NCs

The aqueous extract of *M. officinalis* was used for the synthesis of Ag_2_Se-NCs from AgNO_3_ and Na_2_SeO_3_ as silver and selenium sources, respectively. After stirring for 6 h, the reaction mixture's color changed from orange to gray, as seen in Fig. 2a. This discoloration is possibly due to the excitation of the silver ion surface’s plasmon resonance in the Ag_2_Se-NCs (García et al. 2020). The existence of Ag_2_Se-NCs was also established by the appearance of an absorption band between 350 and 450 nm with an absorption intensity at 395.61 nm compared with other studies (Fig. 2a) (Delgado-Beleño et al. 2018; García et al 2020; Martinez-Nuñez et al. 2017). In the formation of Ag_2_Se-NCs, first selenium ions are reduced to Se_2_ and then co-precipitation occurs with Ag cations. Thus, with lasting reaction time and the reducing selenium by the biomolecules, the adsorption spectrum reaches a steady-state, indicating the presence of a biomolecular matrix as a stabilizer of Ag_2_Se-NCs (Sidorova et al. 2018).

FTIR analysis was conducted to determine the functional interactions between biomolecules and Ag_2_Se-NCs (Fig. 2b). The spectrum of biosynthesized Ag_2_Se-NCs showed an O–H stretch at 3423.3 cm^−1^ corresponding to hydroxyl groups of biological compounds involved in NPs synthesis (Sytu and Camacho 2018). Notable transmission peaks appeared at 1652.9 and 1396 cm^−1^, indicating the presence of Ag–Se bond in the NPs structure (Chougale et al. 2013; Kalishwaralal et al. 2016).

**Fig. 3:**
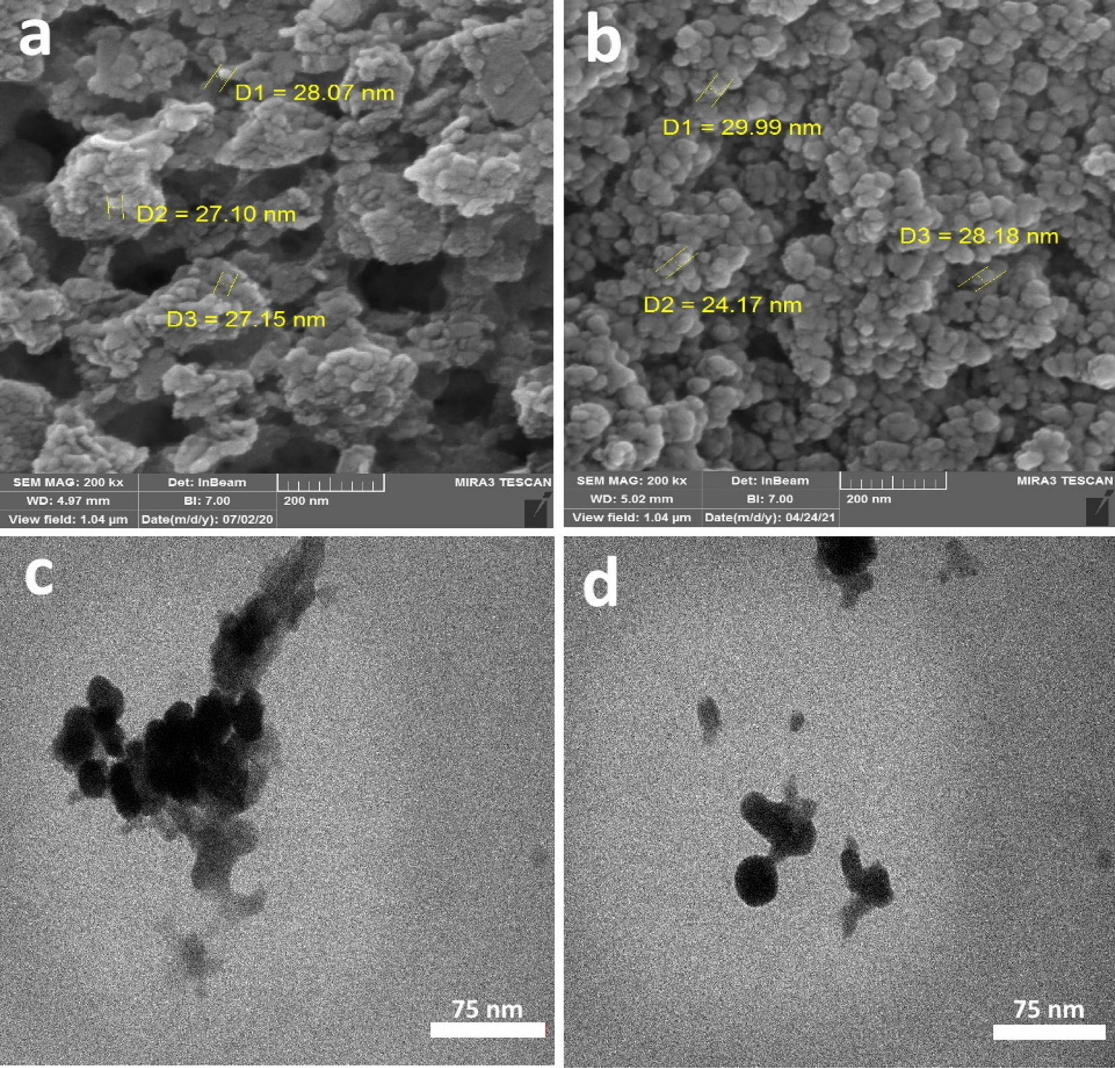
**a**, **b** SEM, **c**, **d** TEM images of biosynthesized Ag_2_Se-NCs

The SEM image shows that the grain of NPs is spherical in shape, about 30 nm, with agglomeration at some places (Fig. 3a, b). Additionally, the TEM image confirmed that the synthesized Ag_2_Se-NCs were spherical with the particle size from 30–40 nm, which agrees with the SEM analysis (Fig. 3c, d).

**Fig. 4:**
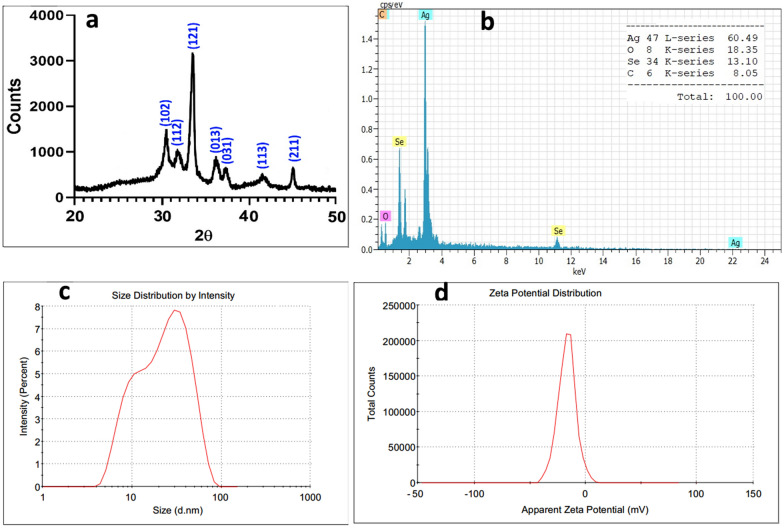
**a** XRD pattern, **b** EDS profile, **c** particle size distribution (DLS) and **d** zeta potential (surface charge) of biosynthesized Ag_2_Se-NCs

As shown in Fig. 4a, the powder XRD patterns represent the formation of β-Ag_2_Se with an orthorhombic crystal structure according to the literature (Ayele 2016; Gholami et al 2018; Sibiya and Moloto 2017). Further, the EDX pattern confirmed the elemental abundance of silver, selenium, oxygen and carbon in the Ag_2_Se nanostructure (Fig. 4b). The size of Ag_2_Se crystals was calculated according to the Scherer equation to be about 38.3 nm, close to SEM and TEM estimations. Additionally, the size distribution curve (DLS) measured a hydrodynamic diameter range of 5–100 nm with a maximum intensity of 51.7 nm in a liquid phase (Fig. 4c).

As seen in Fig. 4d, the zeta potential of Ag_2_Se-NCs in PBS was calculated to be − 15.5-mv. As reported in the literature, NPs with a surface charge of between − 30 and + 30 mV have higher electrostatic stability. On the other hand, bioactive metabolites' role in the stability of NPs was well-established. Therefore, capping agents' existence is crucial to avoid the aggregation of NPs under physiological conditions (Loeschner et al 2011; Vorobyev et al 2018).

### Antibacterial activity studies

**Fig. 5:**
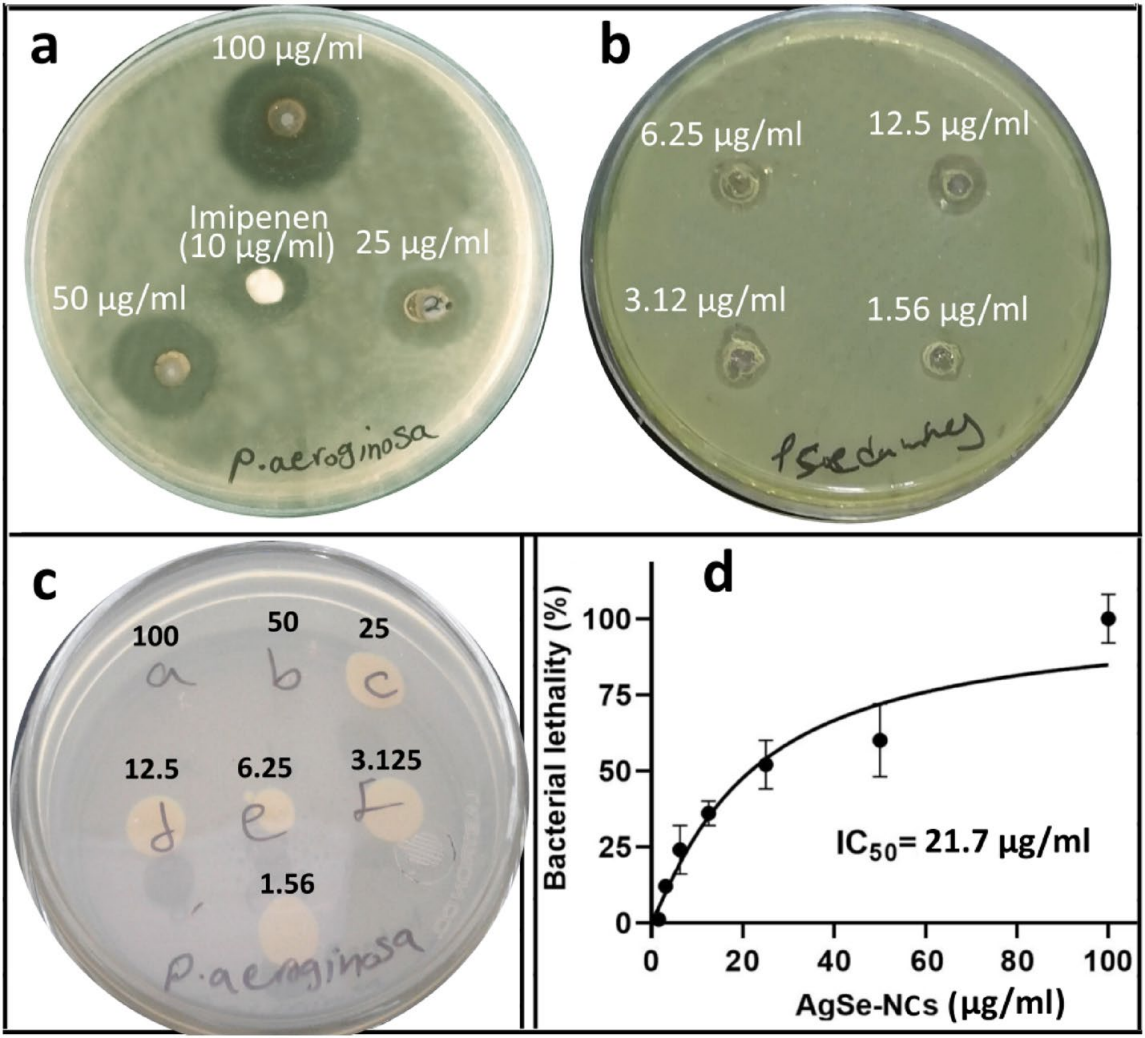
Antimicrobial studies of Ag_2_Se-NCs against *P. aeruginosa* (ATCC 27853) at different concentrations (1.56–100 µg/ml). **a**, **b** Agar well-diffusion assay, **c** MBC on the agar plate and **d** IC_50_ value in terms of MIC results

Antibacterial activity of Ag_2_Se-NCs was measured against *P. aeruginosa* using WDM, MIC and MBC methods. The results showed potent growth inhibition at 100 µg/ml with a MIC and MBC values of 6.25 and 50 µg/ml, respectively (Fig. 5a–c). In this regard, Garcia et al. (2020) have shown that sugar-coated Ag_2_Se-NPs have a robust inhibitory effect on various pathogens, especially Gram-positive bacteria. Contrary to our findings, they reported a greater antimicrobial activity against Gram-positive bacteria (García et al 2020). In the green synthesis of metallic NPs, reducing and capping agents are critical factors for their biological properties so that bioactive compounds can modulate the biological functions of biosynthesized NPs. Various bioactive metabolites include glycosides, saponins, polyphenols, flavonoids, coumarins and alkaloid derivatives with antioxidant, antimicrobial, anticancer, antibiofilm and anti-inflammatory properties, have been reported (Liu et al 2018; Mladenović et al. 2016). However, few studies have been performed on the biosynthesis of NPs by *M. officinalis* metabolites. In one study, Sidorova et al. (2018) reported the antibiotic and antimicrobial effects of silver NPs synthesized with aqueous *MO* extract against *E. coli* and *P. aeruginosa* (Sidorova et al 2018).

### Antibiofilm studies

**Fig. 6:**
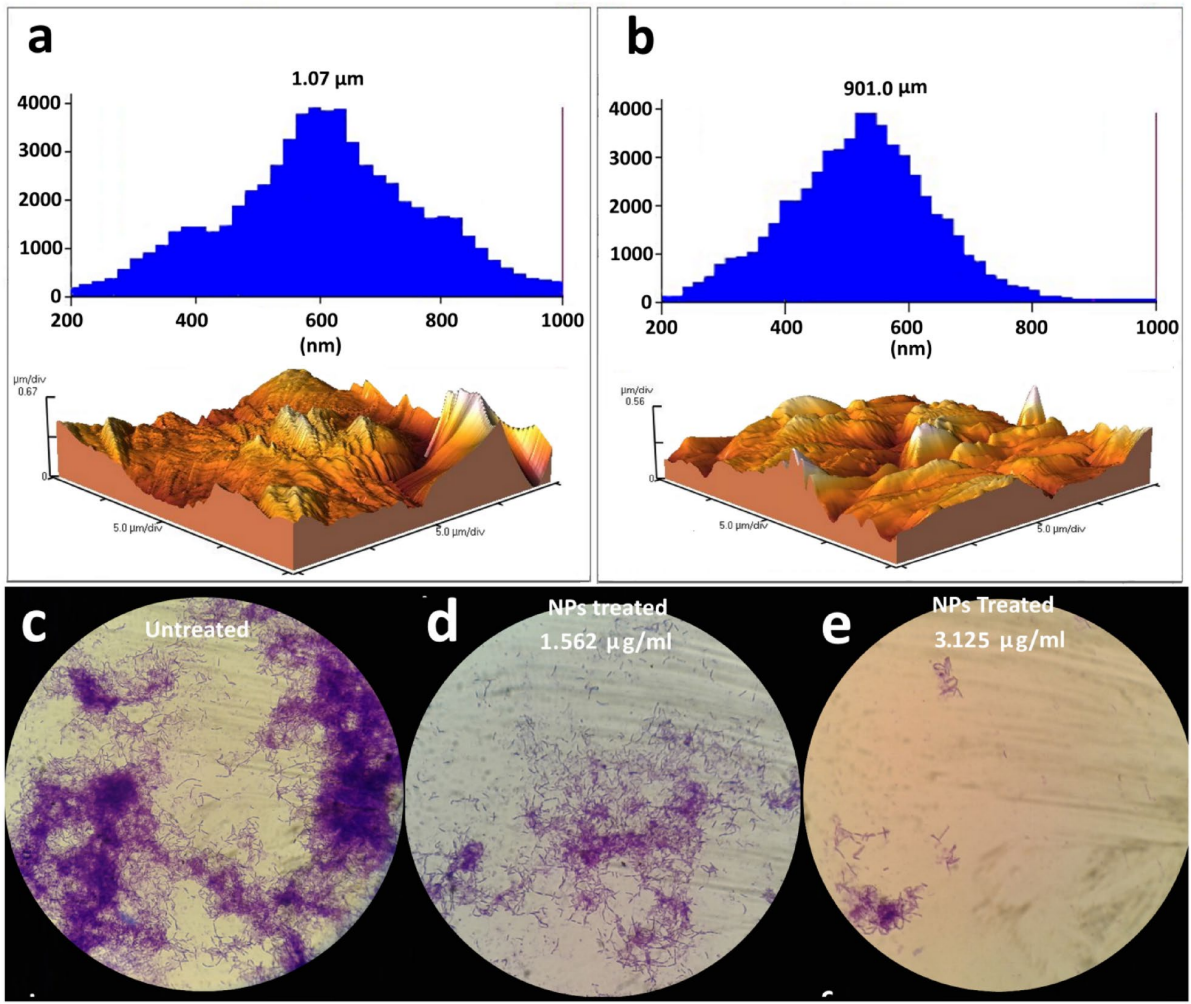
Atomic force microscopy of *P. aeruginosa* biofilm **a** untreated and **b** treated with Ag_2_Se-NCs. Comparative light microscopic images of *P. aeruginosa* biofilm among **c** untreated, **d** treated with 1.56 µg/ml of Ag_2_Se-NCs and **e** treated with 3.125 µg/ml of Ag_2_Se-NCs

Antibiofilm activity assay was performed based on the adhesion ability of *P. aeruginosa* on the glass slide in the presence of different concentrations of Ag_2_Se-NCs. According to the AFM histogram in Fig. 6a, b, the area and height of the biofilm formed by *P. aeruginosa* in the treated and untreated samples are significantly different. The 3D topographic image also confirms the reduction in the level and height of the biofilm (Fig. 6a, b). Furthermore, Fig. 6c–e represents the light microscopic images of biofilm formation by *P. aeruginosa* in the presence of 3.125 and 1.56 µg/ml of Ag_2_Se-NCs. With a qualitative evaluation, biofilm inhibition of Ag_2_Se-NCs is a dose-dependent process, so that with increasing the Ag_2_Se-NCs concentration, the density of the bacterial biofilm was significantly decreased. Many reports have shown that NPs can penetrate bacterial cells and increase its permeability (Subhanandaraj et al. 2020). Metallic NPs bind to biomolecules such as thiol and phosphate groups, disrupting enzyme function and genome integrity (Jiang et al. 2018; Shaikh et al. 2019). The antibiofilm activity of NPs can be due to the inhibition of enzymes involved in the biofilm formation (Gabal et al. 2019; Shah et al. 2019). Qayyum and Khan (2016) suggest that the destruction of the biofilm structure by nanoparticles can sensitize resistant bacteria to antibiotics (Qayyum and Khan 2016).

### Antioxidant activity assessment

**Fig. 7:**
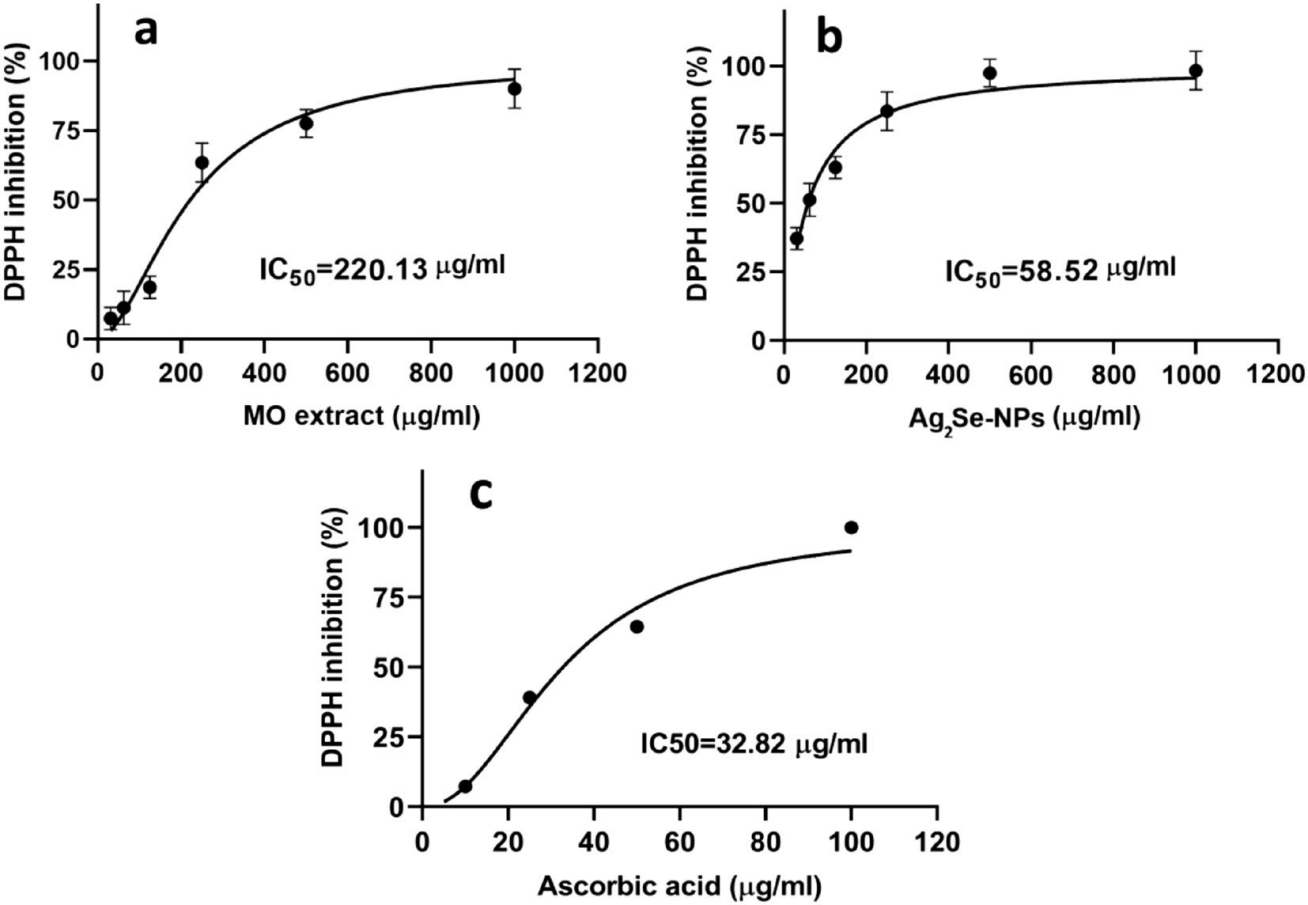
Antioxidant activities of **a** Ag_2_Se-NCs, **b** *MO* extract and **c** ascorbic acid (control)

The antioxidant capacity of Ag_2_Se-NCs, *MO* extract and ascorbic acid, were calculated according to IC_50_, as shown in Fig. 7a–c, respectively. Accordingly, the IC_50_ values calculated for the antioxidant capacity of Ag_2_Se-NCs and MO extract were 58.5 and 220.13 µg/ml, respectively. According to the literature, the antioxidant activity of NPs is probably due to their ability to donate electrons and inhibit free radicals' formation. Ag_2_Se-NCs showed that it has a high potential in scavenging DPPH as a standard model of free radical. Studies show that natural antioxidants can reduce the risk of chronic diseases such as cancer by eliminating free radicals (Kanipandian and Thirumurugan 2014).

On the other hand, studies show that flavonoids and phenolic acids in *M. officinalis* have strong antioxidant capacity. Therefore, potent bioactive compounds can enhance the medicinal properties of biosynthesized NPs (Dobrucka 2020; Liu et al 2018). Since bioactive metabolites in *M. officinalis* extract was confirmed to be strong biological activities and act as capping agents in NPs synthesis, they can exert a synergistic effect on the bioactivity of Ag_2_Se-NCs (Abdelghany et al. 2020; Sytu and Camacho 2018).

### Cytotoxicity effects of Ag_2_Se-NCs

**Fig. 8:**
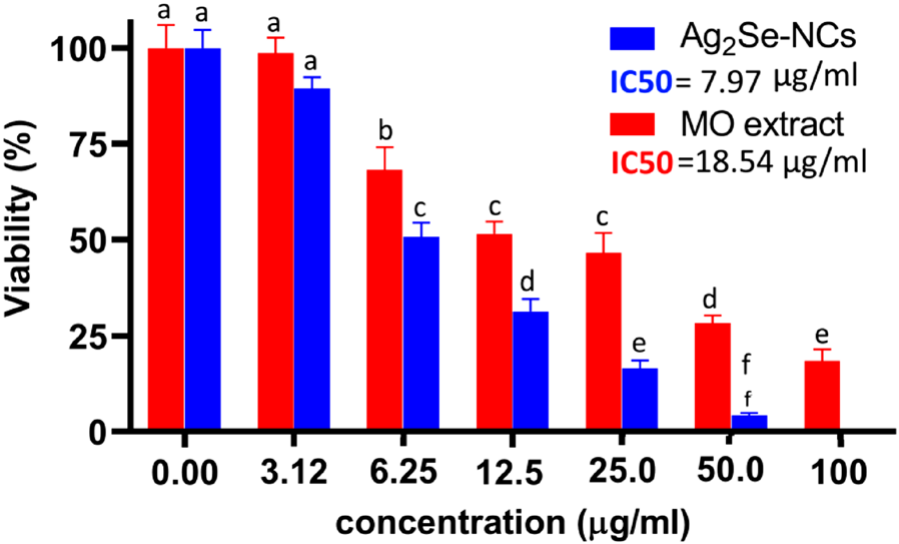
Cytotoxicity of Ag_2_Se-NCs and MO extract in HepG2 cell line. Data are presented as mean ± SD with significant differences of *p* < 0.001. Different superscript shows significant differences between groups

The effect of cytotoxicity of Ag_2_Se-NCs on HepG2 cell line was dose-dependent so that with increasing its concentration, cell survival was significantly reduced. As shown in Fig. 8, the cytotoxicity of Ag_2_Se-NCs was higher than *MO* extract, so that the IC_50_ values of Ag_2_Se-NCs and *MO* extract were 7.97 and 17.44 μg/ml, respectively. Studies have demonstrated that a wide range of mechanisms such as metabolic and structural interactions are involved in the cytotoxicity of NPs (Hemanth Kumar et al. 2019; Reyes-Torres et al. 2019). Kanipandian et al. (2014) evaluated the mechanism of action of cytotoxicity of silver NPs. They concluded that NPs stimulate apoptosis by disrupting cell membrane integrity, inhibiting metabolic pathways, and generating free radicals in the cells (Kanipandian et al. 2014). In this respect, biosynthesized NPs demonstrate more compatibility than chemically synthesized ones so that normal cells do not undergo significant disturbances (Marslin et al. 2018). In the present study, although Ag_2_Se-NCs have more toxicity than the *MO* extract, due to its promising antioxidant properties, it may modulate its toxic effects and increase its biocompatibility (Alves et al. 2019).

## Conclusion

Ag_2_Se-NCs were first successfully synthesized as a reducing and capping agent with *M. officinalis* extract. The results showed that biosynthesized Ag_2_Se-NCs show promising antimicrobial, antibiotic, antioxidant and cytotoxic activities. It really should be mentioned that almost no attention has been paid in earlier studies to the biological properties of Ag_2_Se-NCs. In this study, the growth inhibitory and antibiofilm activities of Ag_2_Se-NCs against antibiotic-resistant *P. aeruginosa* (ATCC 27853) were found to have satisfactory efficacy. Therefore, owing to the advent of new resistant bacteria, our findings could pave the way for the use of Ag_2_Se-NCs as an antimicrobial agent. Taken together, although the biological function of Ag_2_Se-NCs seems to be satisfactory, more in-depth studies are required to recognize the molecular mechanisms and their possible side effects.

## Data Availability

All data obtained in this study are fully analyzed and presented in the manuscript.

## Abbreviations

Ag_2_Se-NCs: Silver selenide nano-chalcogens
FTIR: Fourier transform infrared spectroscopy
UV–VIS: Ultraviolet–visible
NP: Nanoparticle
MNP: Metallic nanoparticle
W/V: Weight/volume
SEM: Scanning electron microscopy
EDX: Energy dispersive X-ray
DLS: Dynamic light scattering
*MO*: *Melilotus officinalis*
ZP: Zeta potential
